# Maternal Exercise Impacts Offspring Metabolic Health in Adulthood: A Systematic Review and Meta-Analysis of Animal Studies

**DOI:** 10.3390/nu15122793

**Published:** 2023-06-19

**Authors:** Lu Ding, Jieying Liu, Liyuan Zhou, Xinhua Xiao

**Affiliations:** 1 Key Laboratory of Endocrinology of National Health Commission, Diabetes Research Center of Chinese Academy of Medical Sciences, Department of Endocrinology, Peking Union Medical College Hospital, Peking Union Medical College, Chinese Academy of Medical Sciences, Xin-Hua Xiao, No. 1 Shuaifuyuan, Wangfujing Street, Dongcheng District, Beijing 100730, China; pumc_luding@student.pumc.edu.cn (L.D.); liujieying50@pumch.cn (J.L.); zhouliyuan_mail@163.com (L.Z.); 2 Department of Medical Research Center, Peking Union Medical College Hospital, Chinese Academy of Medical Sciences & Peking Union Medical College, Beijing 100730, China

**Keywords:** maternal exercise, offspring health, obesity, glucose metabolism, adulthood

## Abstract

Maternal exercise benefits offspring’s metabolic health with long-term repercussions. Here, we systematically reviewed the effects of maternal exercise on offspring obesity outcomes in adulthood. The primary outcome is body weight. The secondary outcomes are glucose and lipid profiles. Two independent authors performed a search in the databases PubMed, EMBASE, and Web of Science. A total of nine studies with 17 different cohorts consisting of 369 animals (two species) were included. Study quality was assessed using the SYRCLE risk of bias. The PRISMA statement was used to report this systematic review. The results showed that maternal exercise contributes to improved glucose tolerance, reduced insulin concentration, and lower total cholesterol and low density lipoprotein levels in adult offspring in mice, which are independent of maternal body weight and offspring dietary condition. Additionally, in rats, maternal exercise leads to a higher body weight in adult offspring, which might be attributed to the high-fat diet of offspring after weaning. These findings further support the metabolic beneficial role of maternal exercise on offspring in adulthood, although the issue of translating the results to the human population is still yet to be addressed.

## 1. Introduction

The prevalence of obesity has reached epidemic proportions [1]. Globally, over 1.9 billion adults were either overweight or obese in 2016, as indicated by the World Health Organization [2]. Specifically, the prevalence of obesity in women of reproductive age continues to increase [3,4,5,6]. Obesity in pregnant women not only increases the risk of developing type 2 diabetes (T2D), pre-eclampsia, and preterm delivery [7,8] but also increases the risk of progressing obesity and chronic metabolic diseases later in the lives of their offspring [9,10,11]. Therefore, maternal intervention with the aim of combating metabolic disorders may be a practical way to break the vicious cycle of obesity occurrence over generations.

Exercise is highly recommended throughout the treatment of different kinds of metabolic disorders. According to the US Department of Health and Human Services (DHHS) and American College of Obstetricians and Gynecologists (ACOG), pregnant women without contraindications are recommended to exercise for at least 20–30 min per day or 150 min per week [12,13]. However, only a limited number of pregnant women meet these recommendations [14,15]. Reasons for lacking exercise during pregnancy varied from lack of time and support to knowledge gaps [16]. An important reason is that studies have inconsistently reported the influence of maternal exercise on offspring health. A large number of studies and meta-analyses indicated that maternal exercise is a safe and beneficial way to benefit fetal and child health [17,18,19], while other studies reached negative results regarding the role of maternal exercise on fetal growth and total body fat [20,21,22]. Thus, it is necessary to provide more comprehensive evidence for the public to fully uncover the benefits of maternal exercise on offspring.

Importantly, most metabolic diseases, such as obesity and T2D, typically occur in adulthood. However, most human studies investigated how offspring health in responds to maternal exercise during infancy and childhood [18,23]. Until now, studies investigating the influence of maternal exercise on the metabolic outcome of adult offspring in humans are still lacking due to the long lifespans of humans. Additionally, human intervention is often intermingled with different lifestyle factors, such as diet, smoking, and stress, which are difficult to control during the long run of trials across an entire lifespan. In this case, animal models, which possess an identical genetic background, a much shorter lifespan, and easily controlled dietary and activity conditions, are imperative for investigating the effects of maternal exercise on offspring metabolic health in adulthood.

In the present study, we aim to systematically review the animal studies investigating the effect of maternal exercise on offspring’s metabolic outcomes in adulthood, providing support and paving the way to conduct longer follow-up studies in humans.

## 2. Methods

### 2.1. Data Sources and Searches

This review was pre-registered on the International Prospective Register of Systematic Reviews (PROSPERO) database (registration ID: CRD42023421078). This review was performed and reported according to the Preferred Reporting Items for Systematic Reviews and Meta-analysis (PRISMA) statement [24]. A systematic electronic search was performed in PubMed, Embase, and Web of Science from inception to 22 February 2023 to identify eligible animal studies in English. To avoid missing relevant articles, we searched the keywords constructed for the following concepts: maternal exposure related, exercise related, offspring related, body mass index (BMI) and obesity related, and animal related. The key words used were (“Maternal” OR “Dam” OR “Pregnancy” OR “Weaning” OR “Gravid” OR “Mother” OR “Gestation” OR “Preterm” OR “Intrauterine” OR” in utero”) AND (“Exercise” OR “Acute exercise” OR “Physical activity” OR “Physical inactivity” OR “Exercise movement techniques” OR “Exercise therapy” OR “Sports” OR “Motor Activity” OR “Sedentary lifestyle” OR “Sedentary” OR “Training” OR “Activity” OR “Muscle” OR “Strength”) AND (“Child” OR “Offspring” OR “Fetus” OR “Fetal” OR “Infant” OR “Infancy” OR “Postnatal” OR “Newborn” OR “Toddler” OR “neonatal” OR “Intergeneration” OR “Adult”) AND (“Body weight” OR “Body mass index” OR “BMI” OR “Pediatric obesity” OR “Body composition” OR “Thinness” OR “Underweight” OR “Obesity” OR “Overweight” OR “Nutritional status” OR “Diet”) AND (“Animal” OR “Animal Experimentation” OR “Laboratory Animal Science” OR “Animals, Newborn” OR “models, animal” OR “Animals, outbred strains” OR “Mammals” OR “Animal study” OR “Rodent” OR “Chordata” OR “Invertebrates”). The related literature of the identified articles was reviewed to discover additional studies that were eligible for the present study.

### 2.2. Eligibility Criteria

Animal studies with no limitation in species were included. The primary outcome was body weight in adult offspring. The secondary outcomes include profiles assessing glucose homeostasis and lipid homeostasis: fasting blood glucose (FBG), the area under the curve (AUC) of glucose tolerance test, insulin level, homeostatic model assessment for insulin resistance (HOMA-IR), triglycerides (TG), total cholesterol (TC), high-density lipoprotein (HDL), low-density lipoprotein (LDL), and free fatty acids (FFA). Studies were included if they compared body weight in adult offspring born to exercised mothers. For mice, adulthood is defined as the period spanning 12–24 weeks [25], and for rats, adulthood spans 24–72 weeks [26]. Studies included pregnant mice with pregnancy normal weight (MNW) and pregnancy obesity (MO). MO was defined as a statistically significant higher body weight of experimental dams compared with control dams during pregnancy. Reviews, editorials, conference abstracts, duplicate reports, and articles not reporting the primary outcomes were excluded.

### 2.3. Study Selection

First, the records were de-duplicated using Endnote. Two reviewers (L.D. and J.L.) independently screened titles and abstracts of all studies for eligibility. Afterward, screening of the full text of eligible studies was performed (L.D., J.L. and L.Z.). Any disagreements were resolved by consensus discussions with the third reviewer when necessary (X.X.).

### 2.4. Data Extraction and Quality Assessment

We extracted general information from each of the eligible studies, including the first author and publication year, sex of the offspring, rodent strain, age of the offspring, the type and duration of maternal exercise, dietary condition of the dams and offspring, and litter size adjustment. For data analysis, we extracted means, standard deviations (SDs) or standard errors (SEs), and number of animals (N) of outcomes. A digital screen ruler (Foxit PhantomPDF) was used for reading graphical results. If the relevant data were not available, we contacted the authors for detailed information. The quality assessment was performed according to SYRCLE guidelines, which have been modified to indicate bias in animal studies based on the Cochrane Risk of Bias tool [27]. Data were independently extracted by the author (L.D.) using standard data extraction forms. Another author (J.L.) double-checked the extraction forms for accuracy. Any disagreements were resolved by consensus discussions with the third reviewer when necessary (X.X.).

### 2.5. Statistical Analysis

Statistical analyses were conducted with Review Manager (RevMan, version 5.3), with a *p* < 0.05 considered statistically significant. The random-effect model was used for synthesizing all outcomes. Meta-analyses were performed for outcomes with at least two studies available. Differences were assessed by standard mean differences (SMDs) with 95% confidence intervals (CIs). SDs would be determined from the SEs based on the methodology from the Cochrane Handbook for Systematic Reviews of Interventions when necessary. Statistical heterogeneity was quantified mainly by the *I*^2^ statistic, with *I*^2^ values greater than 50% proving high heterogeneity [28]. Possible publication bias was evaluated by funnel plots when more than 10 studies were included. We performed subgroup analyses for maternal and offspring dietary conditions and for the maternal intervention period. Additionally, we performed sensitivity analysis for the primary outcome by excluding one study at a time to examine the stability of our results.

## 3. Results

### 3.1. Search Results and Characteristic of the Included Studies

**Figure 1:**
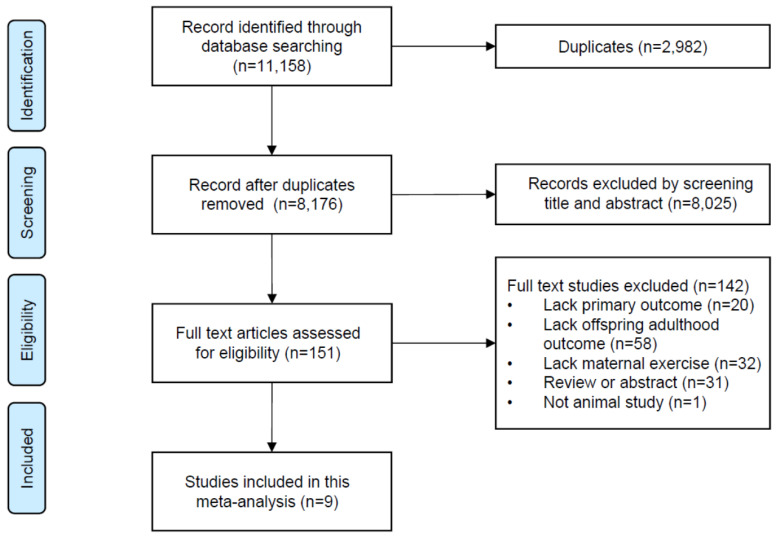
Flow diagram of study selection.

The overview of the search process is shown in Figure 1. Initial searching identified a total of 11,158 records. After removing duplicates and screening the title and abstract, 151 pieces of literature were eligible for full text-screenings. A total of 142 studies were excluded, mainly due to a lack of relevant interventions and outcomes (Figure 1). Eventually, nine studies were included for data extraction [29,30,31,32,33,34,35,36,37]. In total, 369 animals in 17 cohorts across eight outcomes were enrolled in our meta-analysis.

The characteristics of enrolled studies are presented in Table 1. A total of seven studies reported on C57BL/6 mice (*n* = 292) [29,31,32,33,35,36,37], and two studies reported on Sprague–Dawley rats (*n* = 77) [30,34]. Studies included varied exercise types. Most studies intervened with animals with voluntary wheel running. Another study intervened with rats with treadmills. Exercise times also varied, with a range from 3–16 weeks. There are three studies that intervened mice during lactation. Maternal exercise intervention was conducted in females with a higher body weight in five studies, and in females with normal body weight in four studies. Offspring were fed a high-fat diet in four studies and fed a chow diet in five studies. All studies reported the body weight in adult offspring. Most studies reported on glucose homeostasis (*n* = 5), while few studies reported data on lipid profiles (*n* = 2). There are three studies that did not adjust litter size after birth.

### 3.2. Study Quality and Publication Bias

**Figure 2:**
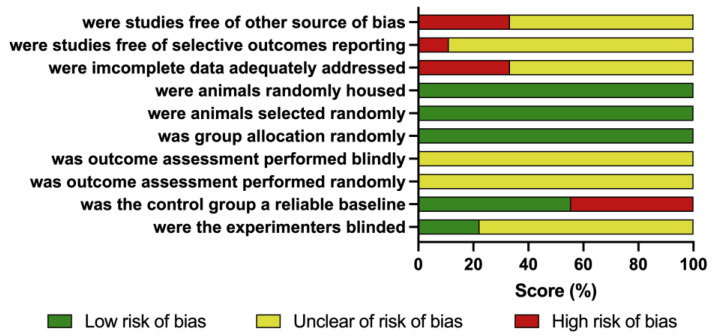
SYRCLE’s risk of bias tool for animal studies. Review authors’ judgments about each risk of bias item presented as percentages across all included studies. Green—low risk, red—high risk, yellow—unclear risk.

The results of the quality assessment of the enrolled studies are summarized in Table S1. Unclear was the most common domain due to a lack of detailed description of the method (50%). A total of two studies reported being blinded during the experiment. A total of three studies did not define the control group with a reliable baseline due to a lack of reporting of litter adjustment. Up to five studies were determined as “low risk of bias” for at least four domains (Figure 2). No evidence of publication bias was detected concerning nine studies with 17 different cohorts in the present study, as the funnel plot was almost symmetrical (Figure S1).

### 3.3. Meta-Analysis Results and Subgroup Analysis

#### 3.3.1. Body Weight

**Figure 3:**
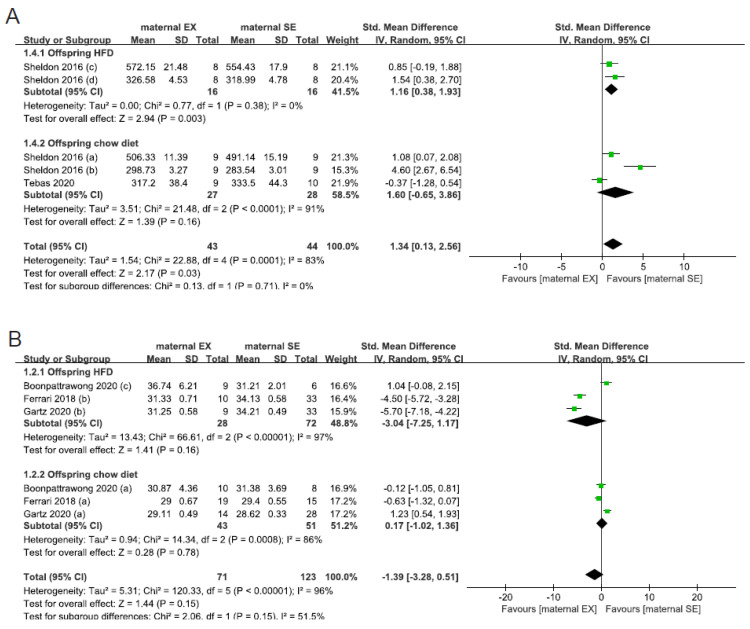

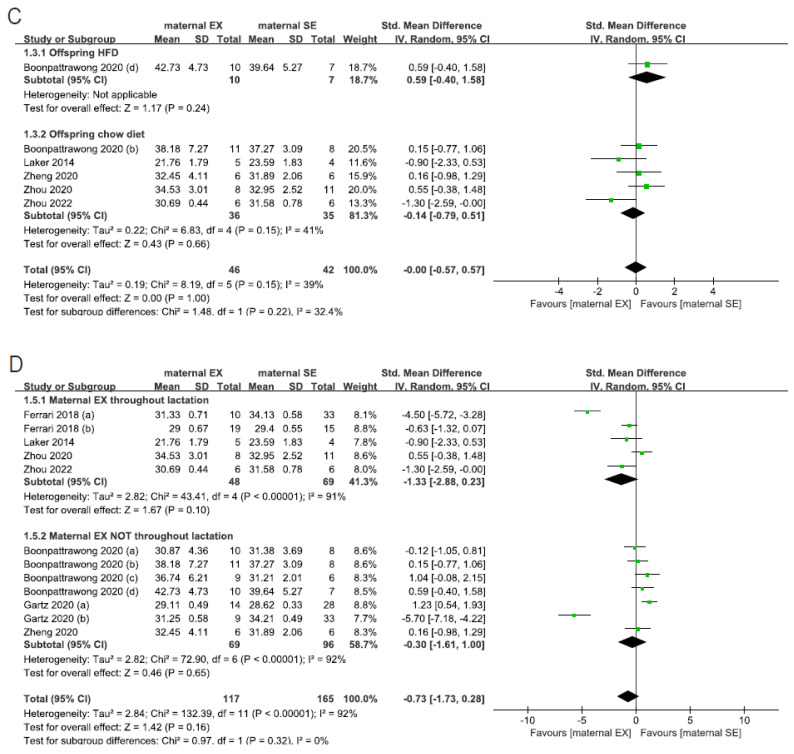
Effect estimates of maternal exercise on offspring obesity outcomes. Subgroup analysis for offspring diet in rats (**A**), for offspring diet of maternal normal weight in mice (**B**), for offspring diet of maternal obesity in mice (**C**), and for maternal exercise throughout (or not) lactation (**D**). EX—exercise, SE—sedentary, HFD—high-fat diet [29,30,31,32,33,34,35,36,37].

Body weight was extracted from nine studies of 369 rodents. The pooled results showed that maternal exercise had no overall significant effect on offspring body weight in adulthood (SMD: −0.11; 95% CI: −0.93, 0.71, *p* = 0.80, *I*^2^ = 91%, Figure S2). However, maternal exercise unexpectedly increased body weight in rats in adulthood (SMD: 1.34; 95% CI: 0.13, 2.56, *p* = 0.03, *I*^2^ = 83%, Figure S2) as species was considered. Further subgroup analysis demonstrated that the increase of body weight in rats might be attributed to offspring high-fat diet (HFD), with a limited number of studies reporting combined male and female data (SMD: 1.14; 95% CI: 0.38, 1.93, *p* = 0.003, *I*^2^ = 0%, Figure 3A). We further separated mice into MNW and MO; however, no significant effect was found in the seven studies (MNW: SMD: −1.39; 95% CI: −3.28, 0.51, *p* = 0.15, *I*^2^ = 96%, Figure 3B; MO: SMD: 0.00; 95% CI: −0.57, 0.57, *p* = 1.00, *I*^2^ = 39%, Figure 3C). Figure 3D showed the result in subgroups of whether there was maternal exposure to exercise during lactation. A trend for decreased body weight in offspring exposed to maternal exercise during lactation was observed, although this did not reach statistical significance (SMD: −1.33; 95% CI: −2.88, 0.23, *p* = 0.10, *I*^2^ = 91%, Figure 3D).

#### 3.3.2. Glucose Homeostasis

**Figure 4:**
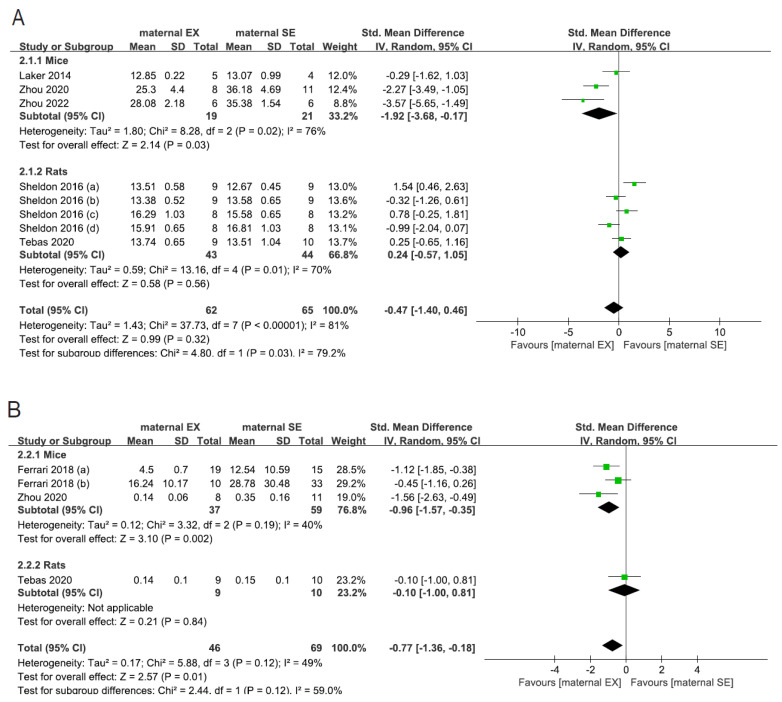
Effect estimates of maternal exercise on offspring glucose homeostasis. Subgroup analysis per species for the AUC of glucose tolerance test (**A**), and for insulin level (**B**). EX—exercise, SE—sedentary, AUC—area under the curve [29,30,31,34,36,37].

A total of five studies with eight different cohorts reported the results of glucose tolerance tests. There was no significant effect of maternal exercise on glucose tolerance in rodents (SMD: −0.47; 95% CI: −1.40, 0.46, *p* = 0.32, *I*^2^ = 81%, Figure 4A). However, in mice, maternal exercise was associated with improved glucose tolerance in studies reporting combined male and female data (SMD: −1.92; 95% CI: −3.68, −0.17, *p* = 0.02, *I*^2^ = 76%, Figure 4A). Additionally, there was no significant effect of maternal exercise on FBG in rodents with a limited number of studies included (SMD: 0.11; 95% CI: −0.60, 0.82, *p* = 0.76, *I*^2^ = 0%, Figure S3).

We extracted data from three studies with four cohorts that investigated the fasting insulin levels under the influence of maternal exercise. Insulin concentration was lower in offspring due to maternal exercise in rodents (SMD: −0.77; 95% CI: −1.36, −0.18, *p* = 0.01, *I*^2^ = 49%, Figure 4B). This effect was mainly dependent upon the species of mice (SMD: −0.96; 95% CI: −1.57, −0.35, *p* = 0.002, *I*^2^ = 40%, Figure 4B) in our data. We did not perform subgroup analysis concerning maternal and offspring dietary condition, intervention duration, and sex due to lack of data.

### 3.4. Lipid Homeostasis

Only two studies reported the effect of maternal exercise lipid homeostasis in male offspring in adulthood. The results showed that maternal exercise significantly reduced TC (SMD: −16.42; 95% CI: −21.25, −11.59, *p* < 0.00001, *I*^2^ = 0%, Figure S4A) and LDL (SMD: −1.49; 95% CI: −2.32, −0.66, *p* = 0.0004, *I*^2^ = 0%, Figure S4C), while no significant difference was reported on TG (SMD: −0.46; 95% CI: −1.19, 0.26, *p* = 0.21, *I*^2^ = 0%, Figure S4B) and FFA (SMD: −1.17; 95% CI: −3.83, 1.49, *p* = 0.39, *I*^2^ = 90%, Figure S4D).

## 4. Discussion

In this study, we systematically reviewed and analyzed the current evidence provided by animal experiments investigating the effect of maternal exercise on offspring’s obesity outcomes in adulthood. Based on the nine studies with 17 different cohorts, 369 animals (two species) were included in the meta-analysis. The results showed that maternal exercise contributes to improved glucose tolerance, reduced insulin concentration, and lower TC and LDL levels in mice adult offspring, independent of maternal body weight and offspring dietary condition. Additionally, in rats, maternal exercise leads to a higher body weight in offspring fed an HFD after weaning

To the best of our knowledge, it is the first meta-analysis investigating the effect of maternal exercise on their offspring’s metabolic outcome in adulthood. The long lifespans of humans and confounding lifestyle factors limited investigation on the offspring’s metabolic health in response to maternal exercise to the period during infancy and childhood [18,19,38,39]. Our finding in mice is consistent with these studies showing that maternal exercise benefits the offspring’s metabolic state. In contrast, we found that maternal exercise had negative impacts on body weight in rats, which might be attributed to the HFD of offspring after weaning. This result is in line with the fact that the offspring’s dietary condition is an important factor for developing obesity and metabolic disorders [1]. Due to the lack of studies reporting the fat differences, we were not able to figure out whether visceral fat and subcutaneous fat of offspring were affected by maternal exercise, respectively. This also suggests a need for further studies to investigate this fat difference, since both fats play roles in developing obesity [40].

Although the number of studies is limited, this meta-analysis further proved the beneficial effect of maternal exercise across the lifespan of F1 offspring to adulthood in mice, adding more comprehensive evidence to support the positive effect of maternal exercise. Since there are still some discrepancies in species, there is a need to follow up with the participants involved in investigating the effect of maternal exercise into adolescence and even adulthood to better predict and uncover the risk of obesity across the lifespan.

### 4.1. Potential Mechanism

Higher body weight is an independent risk factor for developing metabolic disorders, such as glucose tolerance and insulin resistance. However, our finding demonstrated maternal exercise did not alter the body weight in adult offspring in mice. Other mete-analyses investigated the effects of maternal exercise on infancy and childhood in human studies [18,19,38,39]. However, conflicting findings in body weight make it difficult to explain the improved metabolic phenotype of offspring in adulthood. An animal study tracked the mice from exercised mothers over the lifespan to the old stage. They reported that the reduced body weight became statistically significant until 52 weeks of age [41]. On the contrary, the improved glucose tolerance in the offspring from exercised dams precedes decreased body weight [41]. All these findings suggest that the metabolic state of offspring in adulthood induced by maternal exercise might be independent of the alterations in body weight.

Extensive evidence has proved that exercise alters epigenetic modifications, resulting in metabolic benefits [19,42,43]. Meanwhile, epigenetic regulations have been proposed to affect offspring’s metabolic health [44]. Epigenetic alterations include DNA methylation, histone modifications, and microRNA. Currently, studies investigating the epigenetic alterations that occurred during maternal exercise mainly focus on DNA methylation. A study found that maternal exercise resulted in increased placental apelin. Higher levels of apelin activated Tet, which converts 5-methylcytosine in *Prdm16* to 5-hydroxymethylcytosine [45]. Another study reported that maternal exercise prevented *Pgc-1α* hypermethylation, which ameliorated the metabolic dysfunction in offspring [29]. Recently, Kusuyama et al. defined placental superoxide dismutase 3 as an exercise-induced protein that benefits metabolic homeostasis in offspring [46]. It contributed to epigenetic alterations to hepatic metabolic genes by activating the AMPK/TET-signaling axis [46]. Intriguingly, a study found that alterations in DNA methylation induced by maternal intervention were associated with histone post-translational modifications [47], suggesting that the epigenetic modifications of maternal exercise on offspring could be even broader.

### 4.2. Clinical Relevance

It is important to clarify that animal models cannot perfectly represent the human situation, and mice and rats (enrolled species) are nocturnal rodents, which are quite different from humans. Simultaneously, our meta-analysis showed that the effects of maternal exercise on the body weight of offspring in adulthood varied between mice and rats, with a limited number of included studies. Thus, we emphasize the need and the driving force provided by our results to follow up with the participants involved in studies investigating the effect of maternal exercise into adolescence and even adulthood to better predict and uncover the risk of obesity across the lifespan.

In this meta-analysis, we included studies that exposed the animals to intervention during lactation. Studies have proved that HFD exposure during lactation is considered as an independent risk factor for increased offspring body weight compared to restricted HFD exposure during pregnancy [48,49,50]. Meta-analyses also supported a strong correlation between offspring health outcomes and maternal exposure during lactation [51,52]. For humans, the last trimester of gestation is comparable to the lactation in rodents. Consequently, human studies showed that maternal diet intervention in late gestation was correlated with the most evident alterations in neonatal body composition [53]. In our studies, we found a decreased trend in the body weight of offspring exposed to maternal exercise during lactation; however, the data were not statistically significant. This discrepancy also triggers the motivation to conduct a longer follow-up study in humans.

### 4.3. Strengths and Limitations

To the best of our knowledge, ours is the first meta-analysis investigating the effect of maternal exercise on offspring’s metabolic outcome in adulthood in animal studies. It adds evidence to a more comprehensive understanding of the beneficial effects of maternal exercise. Meanwhile, in animal experiments, it makes it possible to exclude different kinds of confounding factors, such as drinking and smoking. Additionally, we conducted an inclusive and broad search of the available evidence to ensure all eligible studies were included.

However, our study possesses several limitations. First, our enrolled studies were performed in mice and rats; both are nocturnal rodents, which are quite different from humans. In such a case, the time and duration of maternal exercise cannot perfectly match that of humans directly. Researchers have reported that larger animal models, such as non-rodent animals, possess a closer condition to human development [54]; future maternal intervention studies in non-rodent animals are urgently needed to provide more solid information to guide human studies. Second, due to a lack of separated data for females and males, we could not perform subgroup analyses in terms of offspring sex. Studies have found sex-dependent differences on offspring in response to maternal interventions [55,56]. However, a meta-analysis showed the negative affects of maternal obesity during pregnancy on offspring cardiometabolic health had nothing to do with offspring sex [57]. Whether maternal exercise exerts sex-dependent effects on offspring metabolic health needs further investigation. Third, the heterogeneity of some results remained high, although we tried to avoid heterogeneity by choosing a random model for meta-analysis. Further, we performed subgroup analyses for maternal body weight, offspring dietary condition, and exposure time during lactation; however, these factors could not explain the heterogeneity. A study reported that the volume of maternal exercise was negatively associated with birth weight [18]; this might be one of the factors that can explain the variation. Last, most animal experiments lacked detailed descriptions of the methodology, which hindered the reliability of quality assessments. Therefore, consistent guideline regarding the experimental details is urgently needed in future animal studies.

## 5. Conclusions

Overall, our findings suggest that maternal exercise contributes to improved glucose tolerance, reduced insulin concentration, and lower TC and LDL levels in adult offspring in mice, which is independent of maternal body weight and offspring dietary condition. In contrast, maternal exercise leads to a higher body weight in rats in adulthood, which might be attributed to the HFD after weaning. These findings further support the metabolic beneficial role of maternal exercise on offspring, although the issue of translating the results to the human population is still to be addressed.

## Figures and Tables

**Table 1:** Overview of studies included in the meta-analysis.

					Maternal Intervention				Offspring Outcome			
Study First Author, Year	N	Sex ^1^	Rodent Strain	Age ^2^	Higher Weight before Exercise	Exercise Type	Exercise Time	HFD Challenge	Bodyweight	Glucose Homeostasis	Lipid Profile	Litters Adjustment
Zhou, 2022 [37]	12	M	C57BL/6	24 weeks	Yes	Voluntary wheel running	3 weeks before mating and throughout pregnancy (6 weeks)	No	Yes	Yes	Yes	Yes
Zhou, 2020 [36]	19	M	C57BL/6	24 weeks	Yes	Voluntary wheel running	3 weeks before mating and throughout pregnancy (6 weeks)	No	Yes	Yes	Yes	Yes
Zheng, 2020 [35]	12	M	C57BL/6	20 weeks	Yes	Voluntary wheel running	3 weeks before mating and throughout pregnancy and lactation (9 weeks)	No	Yes	No	No	Yes
Tebas, 2020 [34]	19	F	Sprague-Dawley rats	25 weeks	No	Treadmill	4 weeks before mating and throughout pregnancy (7 weeks)	No	Yes	Yes	No	Yes
Boonpattrawong, 2020 [33]	69	M	C57BL/6	14 and 17 weeks	Yes	Voluntary wheel running	1 weeks before mating and throughout pregnancy and lactation (7 weeks)	Yes	Yes	No	No	No
Gartz, 2020 [32]	84	M	C57BL/6	16 weeks	No	Voluntary wheel running	9–10 weeks before mating and throughout pregnancy and lactation (15–16 weeks)	Yes	Yes	No	No	Yes
Ferrari, 2018 [31]	77	M	C57BL/6	16 weeks	No	Voluntary wheel running	throughout pregnancy (3 weeks)	Yes	Yes	Yes	No	Yes
Sheldon, 2016 [30]	68	F/M	Sprague-Dawley rats	32 weeks	No	Voluntary wheel running	throughout pregnancy (3 weeks)	Yes	Yes	Yes	No	No
Laker, 2014 [29]	9	F	C57BL/6	24 weeks	Yes	Voluntary wheel running	6 weeks before mating and throughout pregnancy (9 weeks)	No	Yes	Yes	No	No

^1^ M, male; F, female. ^2^ age of exposure to test.

## Data Availability

Data sharing is not applicable.

## Supplementary Materials

The following supporting information can be downloaded at: https://www.mdpi.com/article/10.3390/nu15122793/s1, Figure S1: Publication bias; Figure S2: Forest plot of subgroup analysis per species for body weight; Figure S3: Forest plot of subgroup analysis per species for fasting blood glucose; Figure S4: Forest plot of (A) total cholesterol, (B) triglyceride, (C) low density lipoprotein, and (D) free fatty acids. Table S1: Study quality_risk assessment.
