# Supplementary material for: Maternal Exercise Impacts Offspring Metabolic Health in Adulthood: A Systematic Review and Meta-Analysis of Animal Studies
Source: Nutrients. 2023 Jun 19;15(12):2793. doi: 10.3390/nu15122793 (PMC10303375; doi:10.3390/nu15122793)

Supplementary Materials

1. Supplementary Tables

Table S1. Study quality assessment (.xlsx)

2. Supplementary Figures

Figure S1. Publication bias.

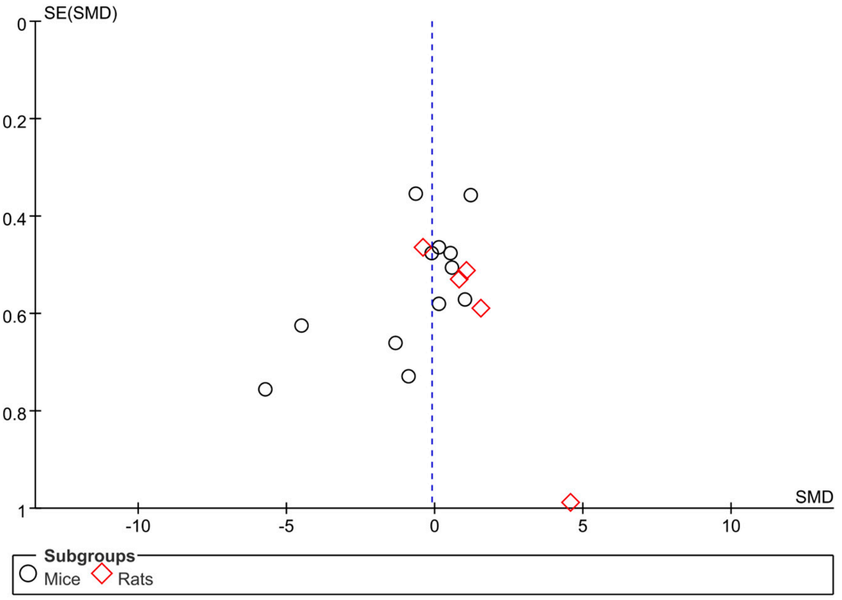

Figure S2. Forest plot of subgroup analysis per species for body weight.

Study or Subgroup	maternal EX Mean	SD	Total	maternal SE Mean	SD	Total	Weight	Std. Mean Difference IV, Random, 95% CI
**1.1.1 Mice**								
Boonpattrawong 2020 (a)	30.87	4.36	10	31.38	3.69	8	6.1%	-0.12 [-1.05, 0.81]
Boonpattrawong 2020 (b)	38.18	7.27	11	37.27	3.09	8	6.1%	0.15 [-0.77, 1.06]
Boonpattrawong 2020 (c)	36.74	6.21	9	31.21	2.01	6	5.9%	1.04 [-0.08, 2.15]
Boonpattrawong 2020 (d)	42.73	4.73	10	39.64	5.27	7	6.0%	0.59 [-0.40, 1.58]
Ferrari 2018 (a)	29	0.67	19	29.4	0.55	15	6.3%	-0.63 [-1.32, 0.07]
Ferrari 2018 (b)	31.33	0.71	10	34.13	0.58	33	5.8%	-4.50 [-5.72, -3.28]
Gartz 2020 (a)	29.11	0.49	14	28.62	0.33	28	6.3%	1.23 [0.54, 1.93]
Gartz 2020 (b)	31.25	0.58	9	34.21	0.49	33	5.4%	-5.70 [-7.18, -4.22]
Laker 2014	21.76	1.79	5	23.59	1.83	4	5.5%	-0.90 [-2.33, 0.53]
Zheng 2020	32.45	4.11	6	31.89	2.06	6	5.9%	0.16 [-0.98, 1.29]
Zhou 2020	34.53	3.01	8	32.95	2.52	11	6.1%	0.55 [-0.38, 1.48]
Zhou 2022	30.69	0.44	6	31.58	0.78	6	5.7%	-1.30 [-2.59, -0.00]
**Subtotal (95% CI)**			**117**			**165**	**71.2%**	**-0.73 [-1.73, 0.28]**
Heterogeneity: Tau² = 2.84; Chi² = 132.39, df = 11 (P < 0.00001); I² = 92%								
Test for overall effect: Z = 1.42 (P = 0.16)								
**1.1.2 Rats**								
Sheldon 2016 (a)	506.33	11.39	9	491.14	15.19	9	6.0%	1.08 [0.07, 2.08]
Sheldon 2016 (b)	298.73	3.27	9	283.54	3.01	9	4.8%	4.60 [2.67, 6.54]
Sheldon 2016 (c)	572.15	21.48	8	554.43	17.9	8	6.0%	0.85 [-0.19, 1.88]
Sheldon 2016 (d)	326.58	4.53	8	318.99	4.78	8	5.9%	1.54 [0.38, 2.70]
Tebas 2020	317.2	38.4	9	333.5	44.3	10	6.1%	-0.37 [-1.28, 0.54]
**Subtotal (95% CI)**			**43**			**44**	**28.8%**	**1.34 [0.13, 2.56]**
Heterogeneity: Tau² = 1.54; Chi² = 22.88, df = 4 (P = 0.0001); I² = 83%								
Test for overall effect: Z = 2.17 (P = 0.03)								
**Total (95% CI)**			**160**			**209**	**100.0%**	**-0.11 [-0.93, 0.71]**
Heterogeneity: Tau² = 2.65; Chi² = 172.04, df = 16 (P < 0.00001); I² = 91%								
Test for overall effect: Z = 0.26 (P = 0.80)								
Test for subgroup differences: Chi² = 6.62, df = 1 (P = 0.01), I² = 84.9%								

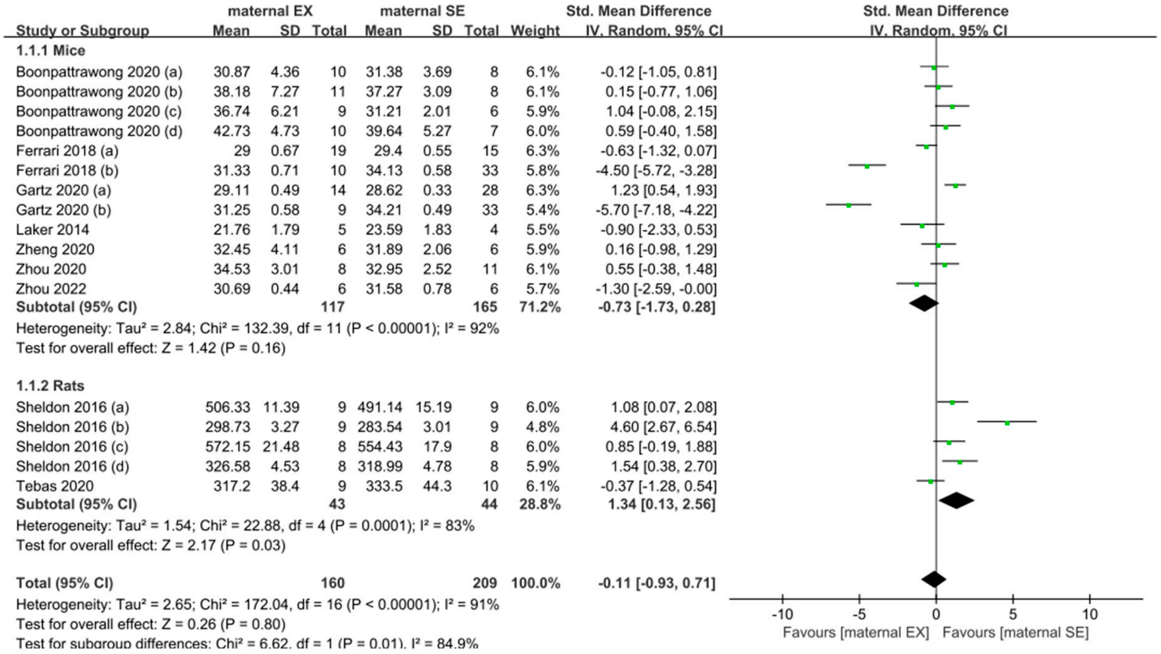

Figure S3. Forest plot of subgroup analysis per species for fasting blood glucose.

Study or Subgroup	maternal EX Mean	SD	Total	maternal SE Mean	SD	Total	Weight	Std. Mean Difference IV, Random, 95% CI
**2.3.1 Mice**								
Zheng 2020	9.26	1.69	6	9.65	2.41	6	39.0%	-0.17 [-1.31, 0.96]
**Subtotal (95% CI)**			**6**			**6**	**39.0%**	**-0.17 [-1.31, 0.96]**
Heterogeneity: Not applicable								
Test for overall effect: Z = 0.30 (P = 0.77)								
**2.3.2 Rats**								
Tebas 2020	5.9	1.1	9	5.66	0.3	10	61.0%	0.29 [-0.61, 1.20]
**Subtotal (95% CI)**			**9**			**10**	**61.0%**	**0.29 [-0.61, 1.20]**
Heterogeneity: Not applicable								
Test for overall effect: Z = 0.63 (P = 0.53)								
**Total (95% CI)**			**15**			**16**	**100.0%**	**0.11 [-0.60, 0.82]**
Heterogeneity: Tau² = 0.00; Chi² = 0.39, df = 1 (P = 0.53); I² = 0%								
Test for overall effect: Z = 0.31 (P = 0.76)								
Test for subgroup differences: Chi² = 0.39, df = 1 (P = 0.53), I² = 0%								

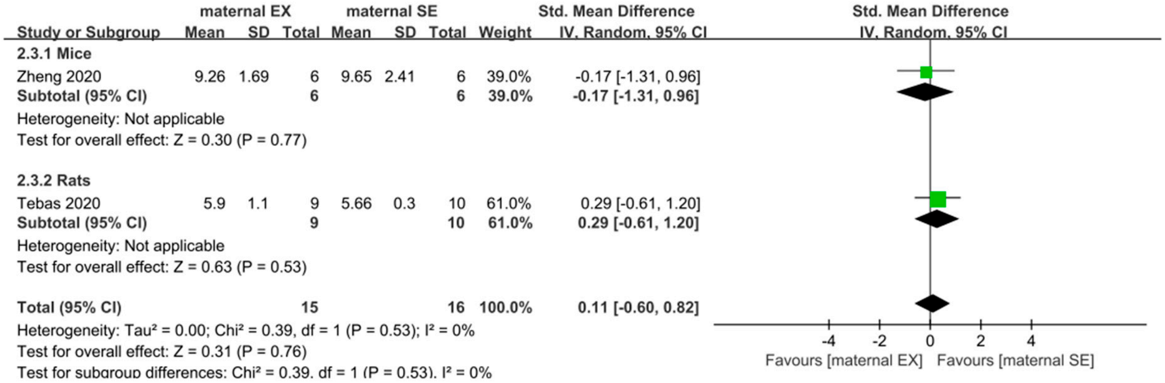

Figure S4. Forest plot of (A) total cholesterol, (B) triglyceride, (C) low density lipoprotein, and (D) free fatty acids.

A

	maternal EX			maternal SE				Std. Mean Difference
Study or Subgroup	Mean	SD	Total	Mean	SD	Total	Weight	IV, Random, 95% CI
Zhou 2020	0.39	0.02	8	2.5	0.14	11	51.7%	-18.64 [-25.36, -11.92]
Zhou 2022	0.4	0.04	6	2.49	0.19	6	48.3%	-14.05 [-21.00, -7.10]
**Total (95% CI)**			**14**			**17**	**100.0%**	**-16.42 [-21.25, -11.59]**

Heterogeneity: Tau² = 0.00; Chi² = 0.86, df = 1 (P = 0.35); I² = 0%
Test for overall effect: Z = 6.66 (P < 0.00001)

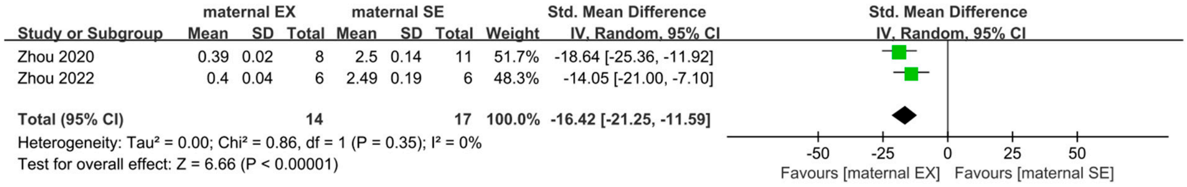

B

	maternal EX			maternal SE				Std. Mean Difference
Study or Subgroup	Mean	SD	Total	Mean	SD	Total	Weight	IV, Random, 95% CI
Zhou 2020	0.39	0.02	8	0.41	0.08	11	62.6%	-0.30 [-1.22, 0.61]
Zhou 2022	0.4	0.04	6	0.45	0.08	6	37.4%	-0.73 [-1.92, 0.46]
**Total (95% CI)**			**14**			**17**	**100.0%**	**-0.46 [-1.19, 0.26]**

Heterogeneity: Tau² = 0.00; Chi² = 0.31, df = 1 (P = 0.58); I² = 0%
Test for overall effect: Z = 1.25 (P = 0.21)

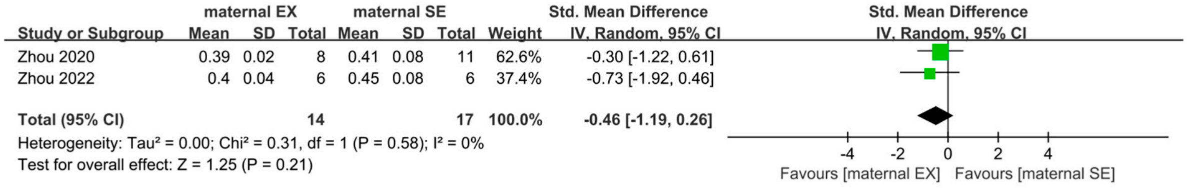

C

	maternal EX			maternal SE				Std. Mean Difference
Study or Subgroup	Mean	SD	Total	Mean	SD	Total	Weight	IV, Random, 95% CI
Zhou 2020	0.11	0.03	8	0.17	0.04	11	60.0%	-1.58 [-2.65, -0.51]
Zhou 2022	0.12	0.03	6	0.19	0.06	6	40.0%	-1.36 [-2.67, -0.05]
**Total (95% CI)**			**14**			**17**	**100.0%**	**-1.49 [-2.32, -0.66]**

Heterogeneity: Tau² = 0.00; Chi² = 0.06, df = 1 (P = 0.80); I² = 0%
Test for overall effect: Z = 3.53 (P = 0.0004)

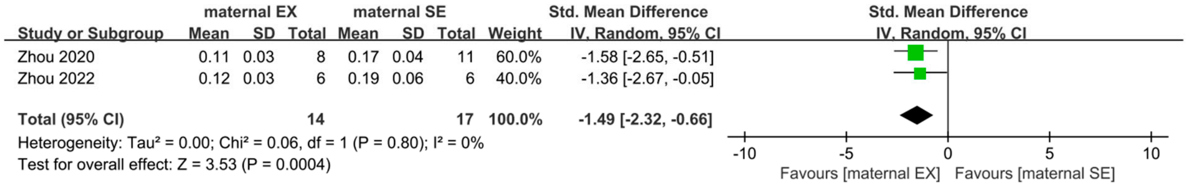

D

	maternal EX			maternal SE				Std. Mean Difference
Study or Subgroup	Mean	SD	Total	Mean	SD	Total	Weight	IV, Random, 95% CI
Zhou 2020	1,272.73	59.34	8	1,447.55	69.58	11	49.3%	-2.55 [-3.83, -1.26]
Zhou 2022	1,350.1	155.8	6	1,311.4	260	6	50.7%	0.17 [-0.97, 1.30]
**Total (95% CI)**			**14**			**17**	**100.0%**	**-1.17 [-3.83, 1.49]**

Heterogeneity: Tau² = 3.30; Chi² = 9.61, df = 1 (P = 0.002); I² = 90%
Test for overall effect: Z = 0.86 (P = 0.39)

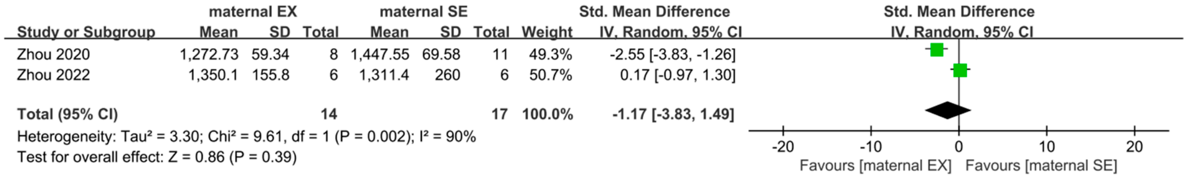

Supplement: Supplementary file 1 [file nutrients-15-02793-s001.zip › Supplemetary materials_proof.pdf]
